# CausalMetaR: An R package for performing causally interpretable meta-analyses – ERRATUM

**DOI:** 10.1017/rsm.2025.22

**Published:** 2025-04-15

**Authors:** Guanbo Wang, Sean McGrath, Yi Lian

Some errors were made during the production of this manuscript and are detailed below.

First, the footnote reads ‘† G.W. and Y.L. equally contributed to this article.’ The correct version of the footnote is: ‘† G.W., S.M., and Y.L. equally contributed to this article.’

Second, there are some typographical errors in the computer code in Table 3 and Section 3.2.2:

1. “source:model” should read “source_model”. There are three instances this (one in Table 3, two in Section 3.2.2)
2. “source:model_args” should read “source_model_args”. There is one instance of this in Table 3.

The publisher apologises for these errors.
